# Integrated multi-omics analyses reveal the TM4SF family genes with prognostic and therapeutic relevance in hepatocellular carcinoma

**DOI:** 10.18632/aging.205398

**Published:** 2024-01-10

**Authors:** Qiang Tang, Shurui Wang, Huimin Li, Junzhi Liu, Xin Hu, Dong Zhao, Maojun Di

**Affiliations:** 1 Department of Gastrointestinal Surgery, Shiyan Taihe Hospital, Hubei University of Medicine, Hubei Province, China; 2 The Second Affiliated Hospital, Zhejiang University School of Medicine, Zhejiang, China; 3 School of Nursing, Peking Union Medical College, Beijing, China; 4 Tianjin Medical University Cancer Institute and Hospital, Tianjin, China; 5 Department of Laboratory Medicine, Zhongnan Hospital of Wuhan University, Wuhan, China; 6 Department of Respiratory and Critical Care Medicine, Renmin Hospital of Wuhan University, Wuhan, China

**Keywords:** TM4SFs, expression, prognostic, tumor-infiltrating immune, therapeutic target

## Abstract

TM4SF family members (TM4SFs) have been shown to be aberrantly expressed in multiple types of cancer. However, a comprehensive investigation of the TM4SFs has yet to be performed in LIHC. The study comprehensively investigated the expression and prognostic value of TM4SFs. Then, a TM4SFs-based risk model and nomogram were constructed for prognostic prediction. Finally, functional loss of TM4SFs was performed to verify the potential role of TM4SFs in LIHC. We found that TM4SFs were significantly up-regulated in LIHC. High expression and hypomethylation of TM4SFs were associated with poor prognosis of LIHC patients. Then, a TM4SFs-based risk model was constructed that could effectively classify LIHC patients into high and low-risk groups. In addition, we constructed a prognostic nomogram that could predict the long-term survival of LIHC patients. Based on immune infiltration analysis, high-risk patients had a relatively higher immune status than low-risk patients. Moreover, the prediction module could predict patient responses to immunotherapy and chemotherapy. Finally, loss-of-function studies showed that TM4SF4 knockdown could substantially suppress the growth, migratory, and invasive abilities of LIHC cells. Targeting TM4SFs will contribute to effective immunotherapy strategies and improve the prognosis of liver cancer patients.

## INTRODUCTION

Hepatocellular carcinoma is one of the most common types of malignancies [1]. Although many advances in clinical and experimental research on LIHC have been made [2], the mechanisms underlying LIHC carcinogenesis and progression remain largely unknown. Recently, surgical resection has been the most effective treatment for patients with LIHC. However, the prognosis of these patients is dismal because of delayed diagnosis, intrahepatic metastasis, and recurrence, with a postsurgical 5-year survival of less than 20% [3].

TM4SF proteins are encoded by the tetraspanin superfamily genes, which are characterized by four highly conserved transmembrane domains (TM1–4), including two extracellular loops and an intracellular loop [4]. These transmembrane proteins are mainly located in the plasma membrane and are required for transmitting signals between the internal or external microenvironments [5]. The transmembrane 4 L6 superfamily (TM4SF) spans over 200-300 amino acids, yielding a molecular size of 20-30 kDa. Several studies have demonstrated that TM4SF1 overexpression positively correlates with tumor grade and can increase the migration and invasion of tumor cells, including ovarian cancer, bladder cancer, and pancreatic cancer [6–8]. TM4SF4 was significantly up-regulated and involved in the maintenance of stemness and epithelial-mesenchymal transition (EMT) in lung cancer [9]. Lee and colleagues showed that TM4SF5 could promote cell adhesion, migration, and invasion by interacting with integrins and actin cytoskeleton remodeling [10]. Furthermore, TM4SF5 could positively regulate EGFR and the classical downstream pathways by activating FAK-c-Src and STAT3 phosphorylation [11, 12]. Eunmi Kim suggested that TM4SF5 expression in MΦs and hepatocytes is critically involved in modulating the inflammatory environment during NAFLD progression [13]. TM4SF18 and TM4SF1 share 60% amino acid sequence homology [14]. Qin reported that TM4SF18 is a promising GC biomarker that promotes the proliferation, migration, and invasion abilities of GC cells and is associated with immune response [15]. TM4SF19, also known as OCTM4, was recognized to be associated with liver fibrosis and carcinomas [16]. The translocation of TM4SF20 has a close relationship with TM4SF4 involvement [17]. The exact role of TM4SFs in the occurrence of LIHC, however, is not very clear. This study set out to systematically identify the prognostic value and clinicopathological features of the TM4SF family gene for LIHC by bioinformatics analyses.

## RESULTS

### Elevated expression of TM4SFs in LIHC

**Figure 1:**
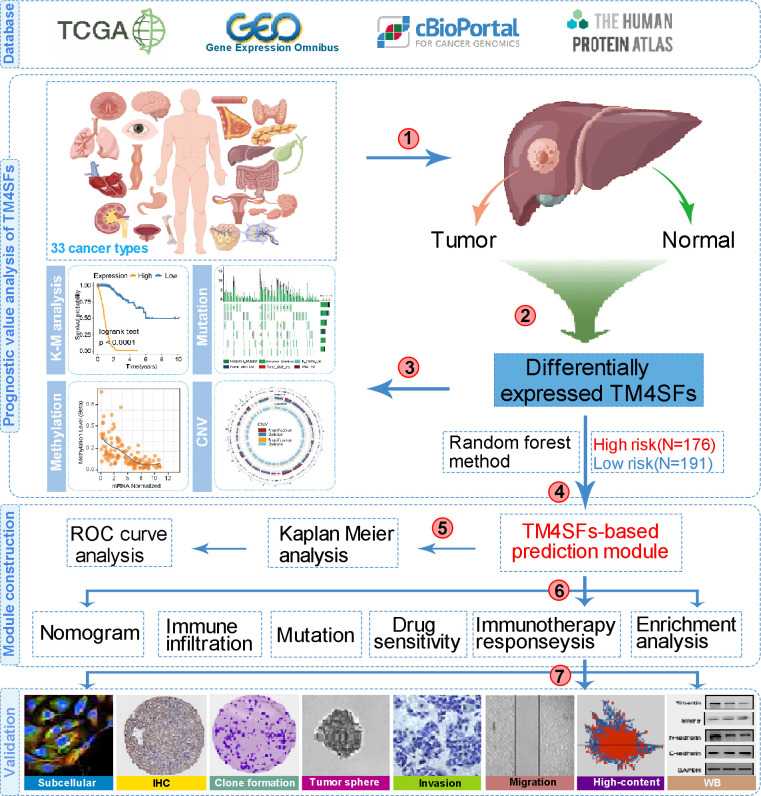
The flow chart of this study.

**Figure 2:**
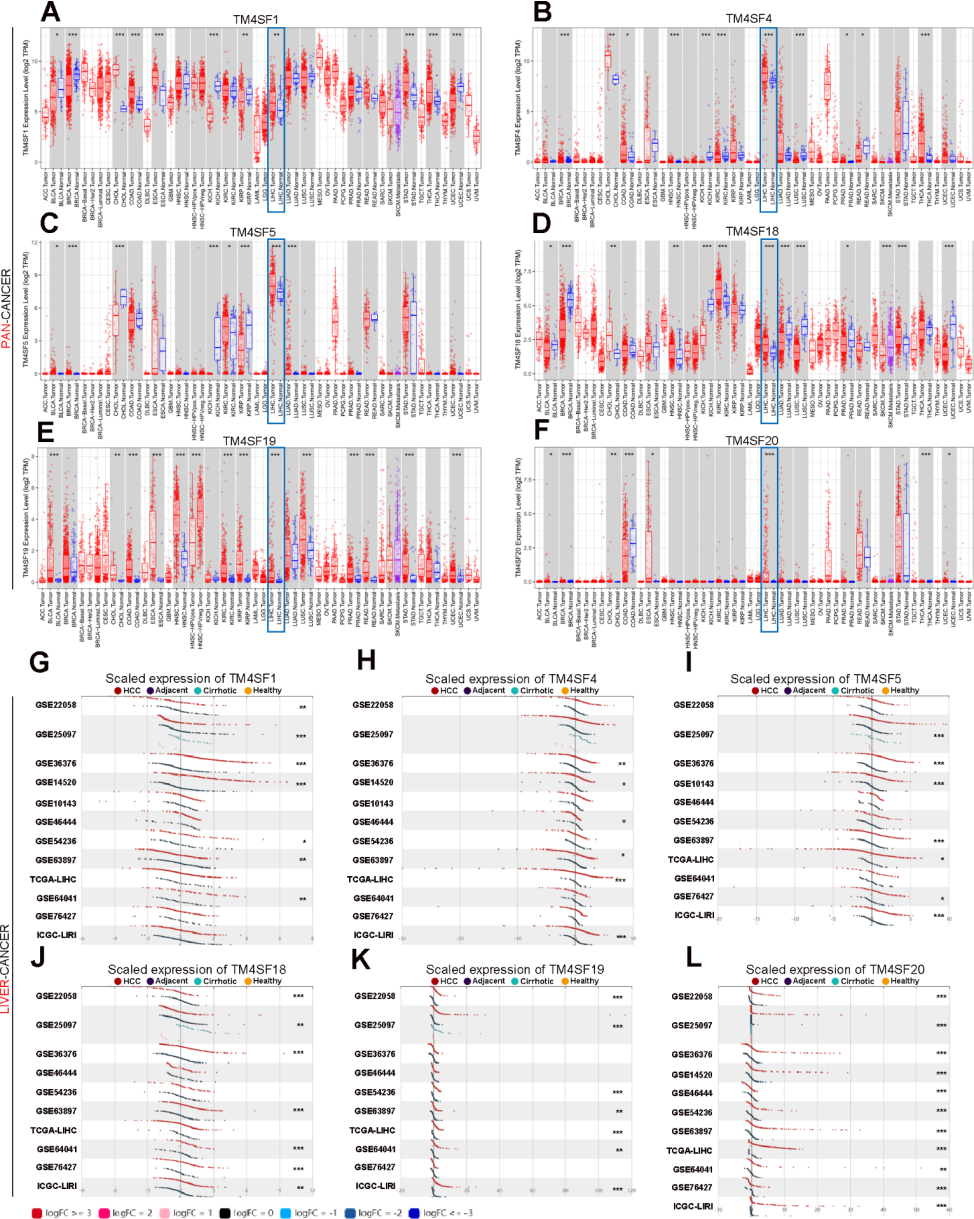
**The transcription levels of TM4SF family members in LIHC.** (**A**–**F**) The expression of TM4SFs in different human tumor types (**T**IMER). (**G**–**L**) Elevated expression of TM4SFs in LIHC (LIHCDB).

The flow chart of the study is shown in Figure 1. We first evaluated the transcription levels of TM4SFs in 33 cancer types by analyzing the TIMER database and found that TM4SFs were abnormally expressed in 33 cancer types from TCGA (Figure 2). TM4SF1 was highly expressed in the majority of tumor types namely, Cholangio carcinoma (CHOL), LIHC, THCA, Esophageal carcinoma (ESCA), and markedly lowly expressed in bladder cancer (BLCA), Kidney renal papillary cell carcinoma (KIRP) (Figure 2A). TM4SF4 was up-regulated in CHOL, COAD, HNSC, PRAD, THCA and markedly lower in BRCA, KICH, KIRC, LUSC, and Rectum adenocarcinoma (READ) (Figure 2B). The expression of TM4SF5 were markedly downregulated in the tissues of in CHOL, KICH, and KIRP (Figure 2C). TM4SF18 was substantially downregulated in BLCA, BRCA, KICH, LUAD, LUSC, PRAD, Skin Cutaneous Melanoma (SKCM) (Figure 2D). TM4SF19 was moderately up-regulated in the tissues of UCEA, KIRP, CHOL, STAD, HNSC, KIRC and PRAD compared with normal adjacent tissues (Figure 2E). And TM4SF20 exhibited a moderate expression in multiple human malignancies (Figure 2F).

We further evaluated the expression level in LIHC using LIHCDB (Integrative Molecular Data base of Hepatocellular Carcinoma, http://lifeome.net/datebase/hccdb/home.html) and found that TM4SF1, TM4SF4, TM4SF5, TM4SF18, and TM4SF19 were all significantly more highly expressed in LIHC from most cases of GEO and TCGA data than in normal adjacent tissues (Figure 2G–2L). In particular, the mRNA expression of TM4SF20 was markedly higher in all GEO microarray datasets than in normal adjacent tissues (Figure 2L). All these results indicated that TM4SFs were highly expressed in LIHC.

### TM4SF overexpression was associated with clinicopathological characteristics in LIHC

Then, we investigated the association between TM4SFs expression and the clinicopathologic characteristics of LIHC, which suggested that TM4SF1 was positively related to advanced pathological grade, lymph node metastasis, and TP53 mutation status (Supplementary Figure 1A). TM4SF4 was markedly associated with race and sex and histological type, which suggested that Caucasian women and patients with fibrolamellar carcinoma displayed markedly higher TM4SF4 expression (Supplementary Figure 1B). The expression of TM4SF5 and TM4SF18 were associated with advanced pathological grade and TP53 mutation (Supplementary Figure 1C). Then, TM4SF19 and TM4SF20 were positively associated with pathological grade and TP53 mutation (Supplementary Figure 1D, 1E). Overall, these results suggested that TM4SF family member expression was markedly associated with clinical tumor progression (grade, stage, histological subtype, and TP53 mutation).

### The prognostic significance for TM4SFs in LIHC

**Figure 3:**
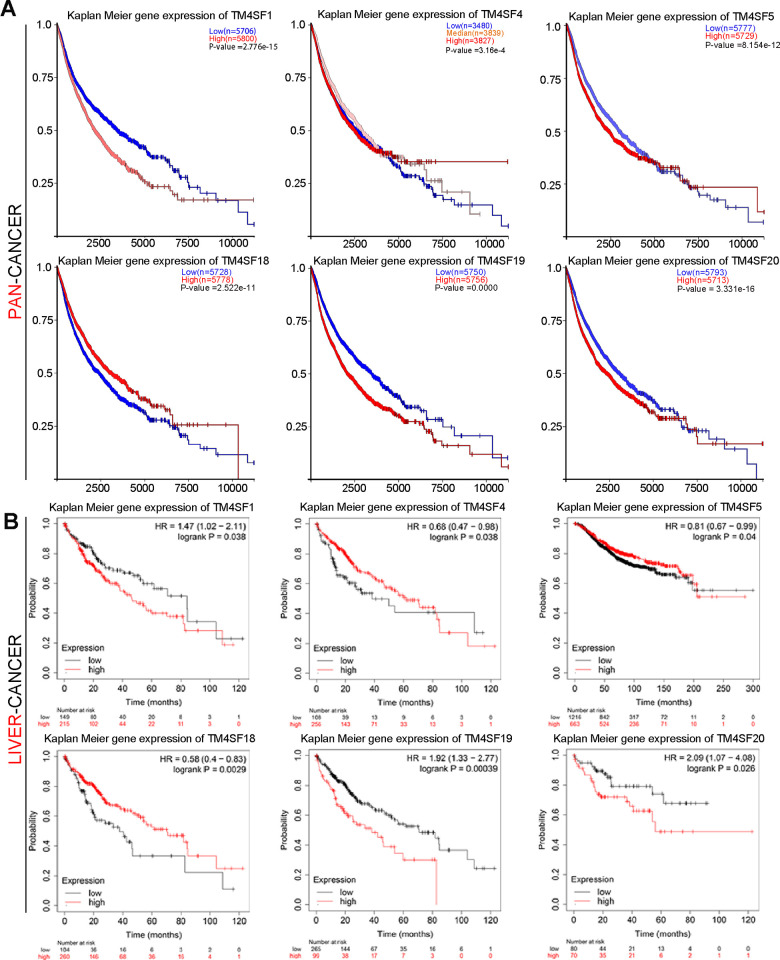
Kaplan-Meier survival curve analysis for TM4SF family members in pan-cancer (**A**) and liver cancer (**B**).

To investigate the prognostic significance for TM4SFs LIHC, Kaplan-Meier plotter was applied. We found that patients with higher expression of TM4SF1, TM4SF5, TM4SF19, and TM4SF20 had shorter survival than those with low expression. In contrast, lower expression of TM4SF18 predicts a poor prognosis in various types of cancers (Figure 3A). From the above data, we found that TM4SFs showed significant prognostic value in cancers. Then, Figure 3. Kaplan-Meier survival curve analysis for TM4SF family members in pan-cancer and liver cancer. We investigate the prognostic significance of TM4SFs in LIHC. The results revealed that higher levels of TM4SF1, TM4SF19, and TM4SF20 were associated with poor OS (Figure 3B), RFS, PPS, and DSS (all P < 0.05). Higher TM4SF20 expression in LIHC was also related to RFS, PPS, and DDS (P < 0.05). In contrast, lower expression of TM4SF4, TM4SF5, and TM4SF18 were found to be associated with poor survival (Supplementary Figure 2). These results indicated that the expression of TM4SFs was related to the survival of LIHC patients. Importantly, higher expression of TM4SF1, TM4SF19, and TM4SF20 can be invoked as potential prognostic markers for LIHC.

### Alteration in the frequency of TM4SFs in LIHC patients

**Figure 4:**
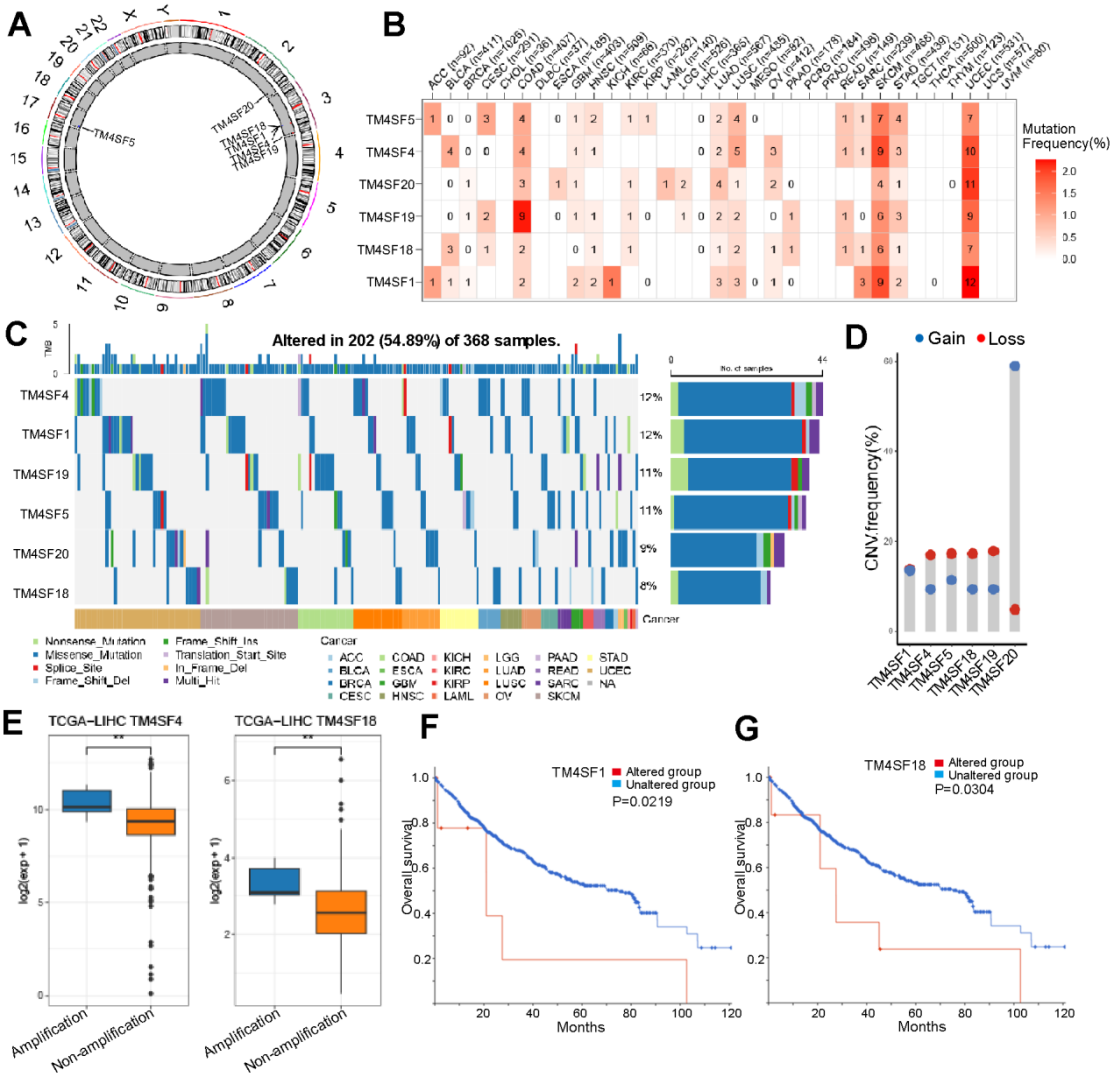
**Different alternation frequencies of TM4SFs and survival analysis in LICH.** (**A**) Chromosomal distributions of TM4SFs are clustered based on their physical locations on the chromosome. (**B**, **C**) Mutation ratio of TM4SFs in LIHC and pan-cancers. (**D, E**) Genetic alteration of TM4SFs in LIHC. (**F**, **G**) Kaplan–Meier plots comparing OS in patients with/without TM4SFs alterations in LICH.

We also analyzed the mutational landscape of the TM4SF family among LIHC patients from the cBioPortal database. The results implied that the mutation frequency involving TM4SFs was not high in most cancers, excluding SKCM, UCEC, and COAD (Figure 4A, 4B). The most common type of genetic alteration was genetic missense mutation in the TM4SF family. The mutation frequencies of TM4SFs were 12%, 12%, 11%, 9%, and 8% (Figure 4C). Somatic copy-number alteration (SCNA) is an essential type of structural variation involved in tumorigenesis and tumor prognosis. Therefore, we further explored the relationship between the SCNA of TM4SF family members. The results suggested that genetic alterations were most frequently identified in TM4SF20, all deep-loss mutations. More specifically, genetic alterations in TM4SF1 only included gain mutations in LIHC (Figure 4D). Moreover, we explored the prognostic impact of TM4SF mutational status on OS in patients with LIHC. The results suggested that patients with TM4SF1 and TM4SF18 mutations exhibited a markedly shorter OS than patients without mutations (Figure 4F, 4G).

### DNA methylation of TM4SFs correlated with prognosis in LIHC

**Figure 5:**
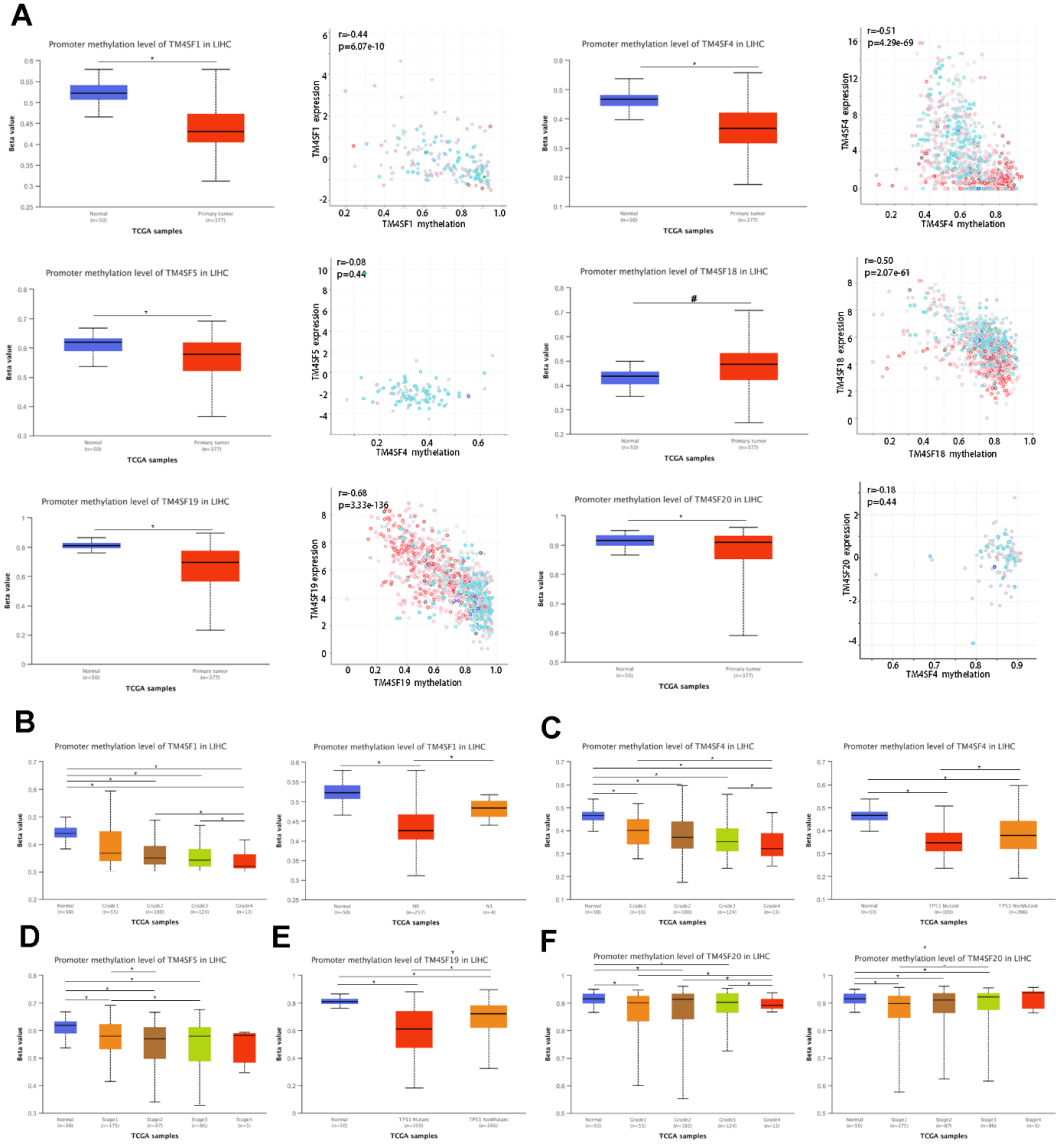
**DNA methylation of TM4SFs expression levels correlated with prognosis in LIHC.** (**A**) The promoter of TM4SFs was hypomethylated in LIHC tissue compared with adjacent normal tissue. (**B**–**F**) The correlation between DNA methylation and the clinical characteristics of LIHC patients.

DNA methylation is the principal epigenetic modification in humans, and changes in methylation levels are regarded as an indication of tumor progression. Low genome methylation is closely related to tumor invasion, metastasis, and prognosis. As shown in the previous subsection, the mutation frequency involving the TM4SFs was low. Therefore, we hypothesized that the hypomethylation events of TM4SFs resulted in gene expression changes. The results demonstrated that the promoters of TM4SF1, TM4SF4, TM4SF5, TM4SF19, and TM4SF20 were hypomethylated in LIHC tissue compared with adjacent normal tissue. The expression levels of the TM4SF1, TM4SF4, TM4SF18, and TM4SF19 correlate inversely with DNA methylation (Figure 5A), which suggests that genes hypomethylated may be directly responsible for the upregulation of these genes in LIHC. Furthermore, we found that the levels of genomic DNA methylation in TM4SFs negatively correlated with tumor grade. More specifically, TM4SF1 methylation was associated with tumor grade and lymph node metastasis (Figure 5B). The methylation levels of TM4SF4 and TM4SF19 were lower in p53 mutant tumors than in wild-type p53 tumors (Figure 5C–5E). In addition, TM4SF20 promoter methylation in tumors was closely associated with tumor stage: stage I tumors in LIHC patients were markedly more likely to have lower methylation levels (Figure 5F). All these results demonstrated that the promoter methylation levels of TM4SFs affect gene expression and are involved in LIHC progression.

CpG methylation data of TM4SF family members were extracted from MethSurv, and the significant prognostic values of CpG in the TM4SFs were investigated by multivariable survival analysis, which suggested that two CpGs of TM4SF1, four CpGs of TM4SF4, three CpGs of TM4SF19, and five CpGs of TM4SF20 were associated with significant prognosis (Table 1). Then, we identified one critical CpG of TM4SF1, two critical CpGs of TM4SF4, two critical CpGs of TM4SF5, and three critical CpGs of TM4SF19. Then, K-M analysis revealed that the critical CpGs were positively correlated with the OS of patients (Supplementary Figure 3A–3H).

**Table 1:** The significant prognostic values of CpG in the TM4SFs family members.

**Gene symbol**	**CpG name**	**Hazard ratio**	**CI**	**P-value**	**UCSC RefGene Group**	**Relation to UCSC CpG island**
**TM4SF1**	cg00244111	0.681	(0.466;0.995)	0.047	3'UTR	Open_Sea
	cg16705300	1.423	(1.003;2.02)	0.048	TSS1500	Open_Sea
**TM4SF4**	cg08667024	0.485	(0.336;0.7)	0.0001	Body	Open_Sea
	cg12874219	2.787	(1.672;4.643)	8.334E-05	Body	Open_Sea
	cg16954508	1.489	(1.054;2.103)	0.024	3'UTR	Open_Sea
	cg19422253	1.894	(1.307;2.745)	0.001	Body	Open_Sea
**TM4SF5**	cg01487803	0.645	(0.449;0.926)	0.018	TSS1500	Open_Sea
	cg14208070	1.496	(1.049;2.132)	0.026	Body	Island
	cg18363008	1.697	(1.196;2.407)	0.003	TSS200	Open_Sea
**TM4SF19**	cg05445326	0.634	(0.44;0.913)	0.014	TSS1500	Open_Sea
	cg13314965	0.66	(0.455;0.958)	0.029	TSS1500	Open_Sea
	cg21090033	0.604	(0.416;0.877)	0.008	TSS200	Open_Sea
	cg22496559	0.632	(0.437;0.913)	0.014	TSS200	Open_Sea
	cg27088176	0.632	(0.436;0.917)	0.016	3'UTR	Open_Sea

### Construction of TM4SFs-based prognostic risk model

**Figure 6:**
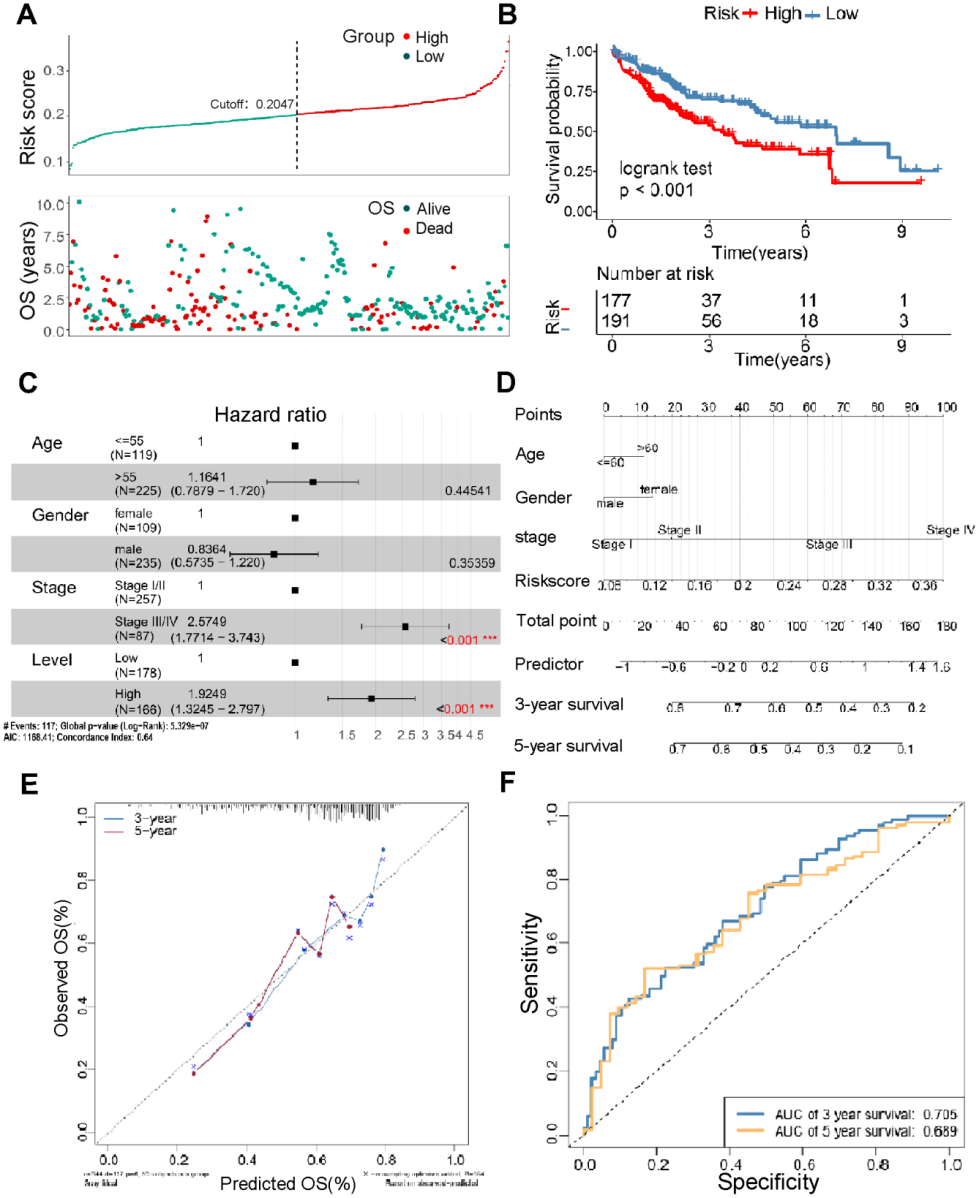
**Construction of the TM4SFs-based risk score for prognostic evaluation.** (**A**) The survival time of LIHC patients with different risk scores. (**B**) The survival curve for the TM4SFs-based risk score of the LIHC cohort in TCGA. (**C**) Multivariable Cox regression analysis demonstrated that the risk models were independent predictors of prognosis for LIHC patients. (**D**) A nomogram was constructed using the multivariable analysis results (age, sex, stage, and risk score) to visualize the model. (**E**) The calibration plots for the 3- and 5-year OS were predicted well in the TCGA cohort. (**F**) Time-dependent ROC curves of the risk score for predicting 3- and 5-year survival rates in the pooled HCC cohort.

To further examine the prognostic value of TM4SFs, we constructed a TM4SF-based model using the random forest method. All patients were divided into low and high-risk groups depending on the risk model. We found that patients in the high-risk group died earlier and had less survival probability than those in the low group (Figure 6A, 6B). Multivariate Cox regression analysis suggested that the risk signature was a novel predictor of prognosis for LIHC patients (HR = 1.92, 95% CI = 1.3–2.8, P < 0.05) (Figure 6C). Then, we constructed nomograms using the multivariable analysis results (age, sex, stage, and risk score) to visualize the model (Figure 6D). In addition, the calibration plots for 3- and 5-year OS were shown in Figure 6E, which suggested that this module had an excellent prediction value in the TCGA cohort. Time-dependent ROC curves showed that the nomogram based on the best model showed good stability for 3 and 5 years survival (Figure 6F).

### Association of the risk signature with immune cell infiltration in LIHC patients

**Figure 7:**
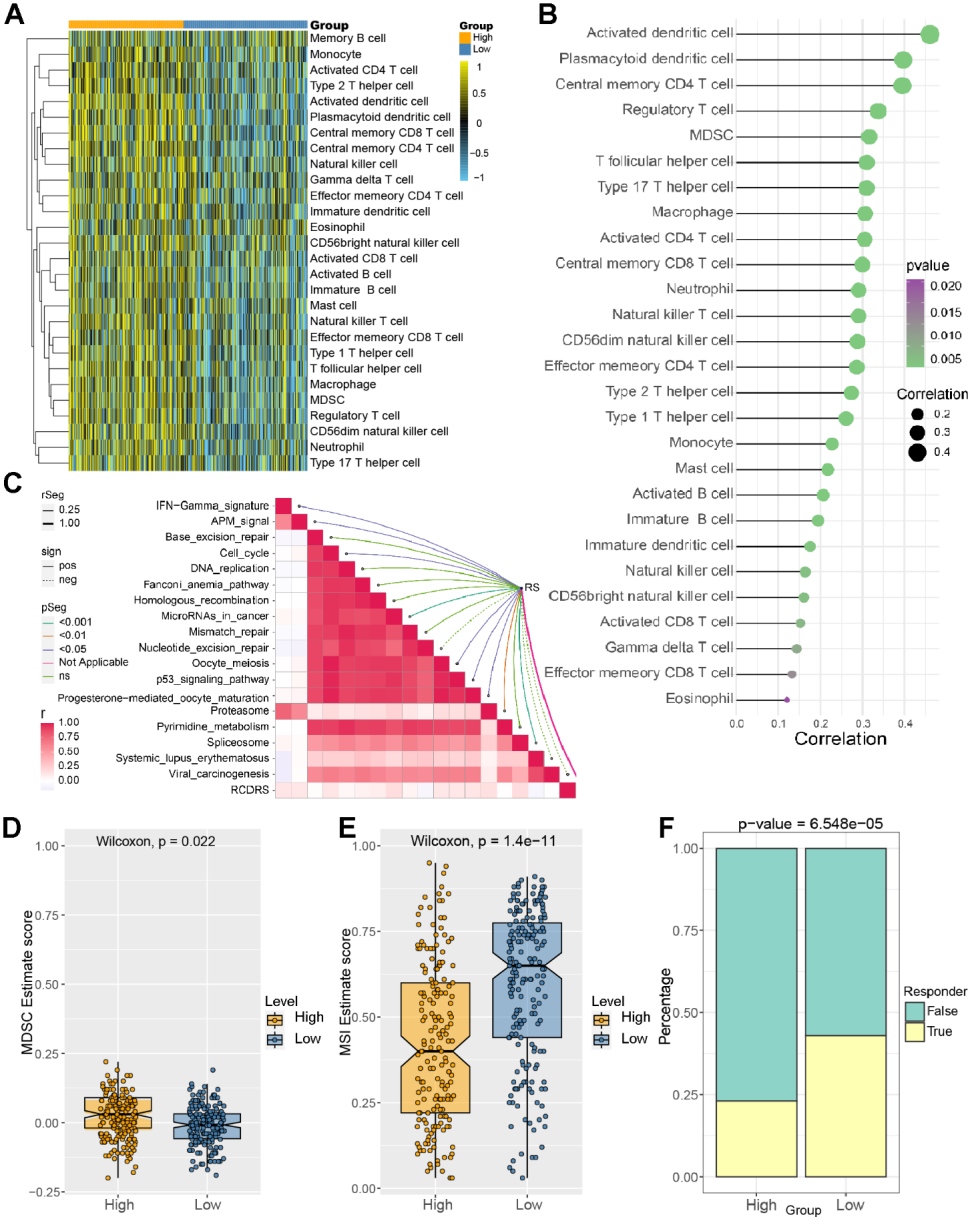
**Correlation between immune cell infiltration level and risk score.** (**A**) Identification of the relative infiltration of 28 types of immune cell subpopulations in the high- and low-risk signature subgroups. (**B**) The correlation between immune infiltration cells and the risk score. (**C**) The relationship between the risk score and immunotherapy-relevant pathways. (**D**, **E**) The risk score was positively correlated with MDSC levels but negatively correlated with the microsatellite instability (MSI) index. (**F**) Evaluation of the TIDE score between the high- and low-risk groups.

**Figure 8:**
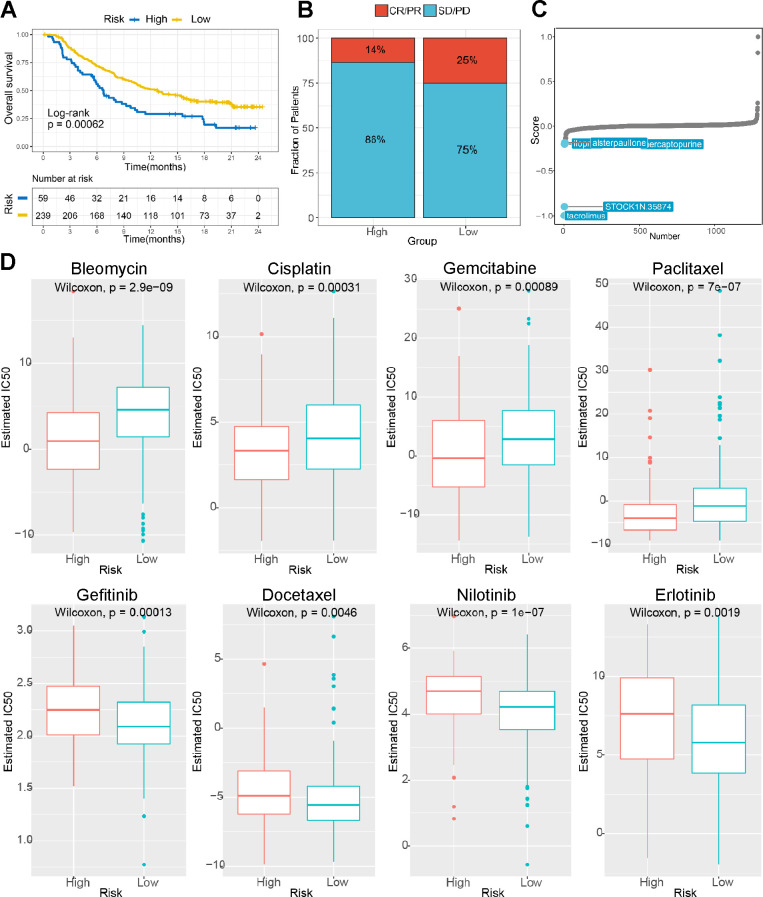
**The risk score predicts sensitivity to immunotherapy and chemotherapy.** (**A**) K-M survival analysis of the patients in the high and low risk subgroups according to the anti-PD-L1 cohort (IMvigor210 cohort). (**B**) The proportion of the immune response to anti-PD-L1 treatment in the high- and low-risk score subgroups. (**C**) Small molecule inhibitor screening based on the prediction model. (**D**) The IC50 values for anticancer drugs, chemotherapeutics, and targeted agents in the high- and low-risk subgroups.

Then, we explore the difference in the proportion of immune cell infiltration between different risk groups. The results suggested that immune cells showed differential infiltration patterns in the different groups. The high-risk group was associated with high immune infiltration status compared to the low-risk group (Figure 7A). In addition, the risk scores were more positively associated with central memory CD4 T cells, regulatory T-cell MDSCs, and type 17 T helper cells (Figure 7B). These demonstrated that LIHC patients with different risk scores may have different immune statuses and diverse outcomes. Figure 8C shows the association between the risk score and immunotherapy-relevant pathways. Furthermore, we found that the risk score was positively correlated with MDSC levels but negatively correlated with microsatellite instability (MSI) (Figure 7C, 7D).

Then, we analyzed the TIDE score between the different groups, and the results suggested that the low-risk group patients were more sensitive to immune checkpoints. (Figures 7E and 8A, 8B). Figure 8C, 8D showed that our risk score could predict the response to small molecule kinase inhibitors and commonly used chemotherapy drugs. The above findings suggest that tumor mutation load might be a novel indicator for LIHC patients.

### Enrichment analysis of TM4SF family members in LIHC patients

**Figure 9:**
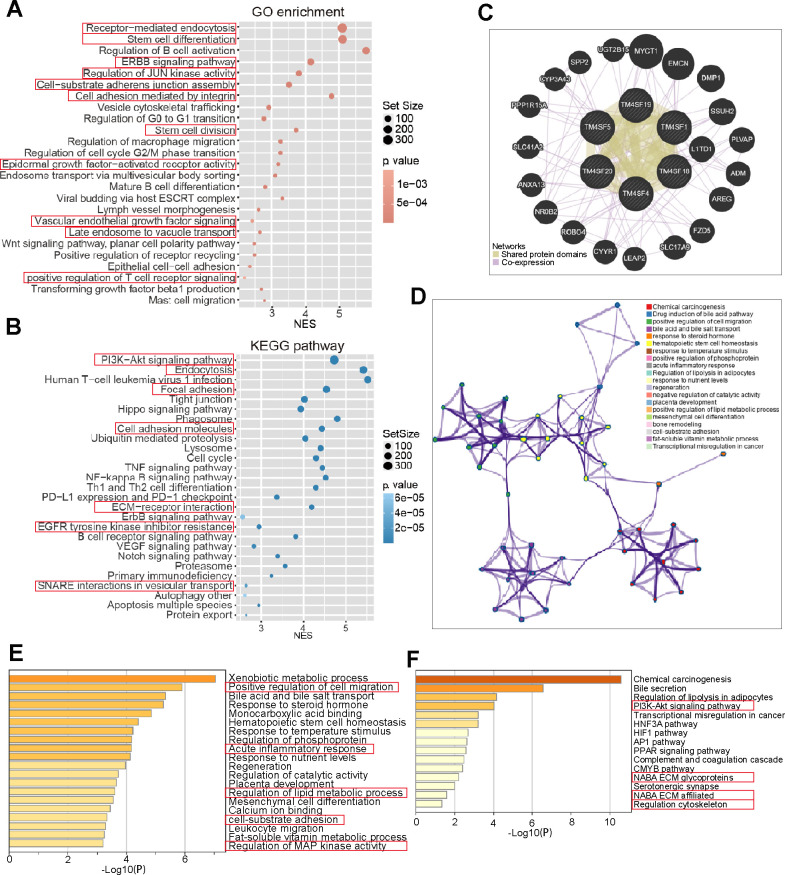
**Gene network and functional enrichment of TM4SFs in LICH.** (**A**, **B**) Risk score-related genes were subjected to GO and KEGG enrichment analyses. (**C**) Construction of a Gene network of TM4SFs and their functionally related genes using GeneMANIA. (**D**) The network of enriched terms was established and colored by ID. (**E**) GO enriched gene pathways and functional analysis of TM4SFs in LIHC. (**F**) KEGG-enriched gene pathways and functions of TM4SFs in LIHC.

We next applied GSEA to investigate the potential biological processes and pathways between the two risk groups. The bubble plot was to visualize enriched GO terms and KEGG pathways as shown in Figure 9A, 9B. GO terms were mainly enriched in receptor-mediated endocytosis, stem cell differentiation, ERBB pathway, cell adhesion mediated by integrin, regulation of G0 to G1 transition, regulation of EGFR activated receptor activity, and mature B-cell differentiation. KEGG analysis revealed that these genes were mainly enriched in the PI3K−Akt pathway, endocytosis, focal adhesion tight junction, cell adhesion molecules, lysosome, cell cycle, ErbB pathway, EGFR inhibitor resistance, and VEGF pathway (Figure 9A, 9B). To further evaluate the biological function of the differentially expressed TM4SFs and the protein interactions and co-expressed genes, a GENE network was constructed to explore the association between the differential expression of TM4SF family genes. We established a network of enriched terms colored by ID. GO and KEGG enrichment analysis results showed that these related genes were mainly enriched in GO:0030335: positive regulation of cell migration, GO:0002526: acute inflammatory response, GO:0031589: cell-substrate adhesion, GO:0043405: regulation of MAP kinase activity; ko05204: chemical carcinogenesis, hsa04151: PI3K-Akt signaling pathway, M255: PID HIF1 pathway, hsa03320: PPAR signaling pathway and so on (Figure 9C–9F). These results showed that the primary functions of TM4SFs were cell adhesion and stemness, the activation of immune cells, the inflammatory response, and EGFR-related pathways.

### The expression of TM4SF family members in LIHC

**Figure 10:**
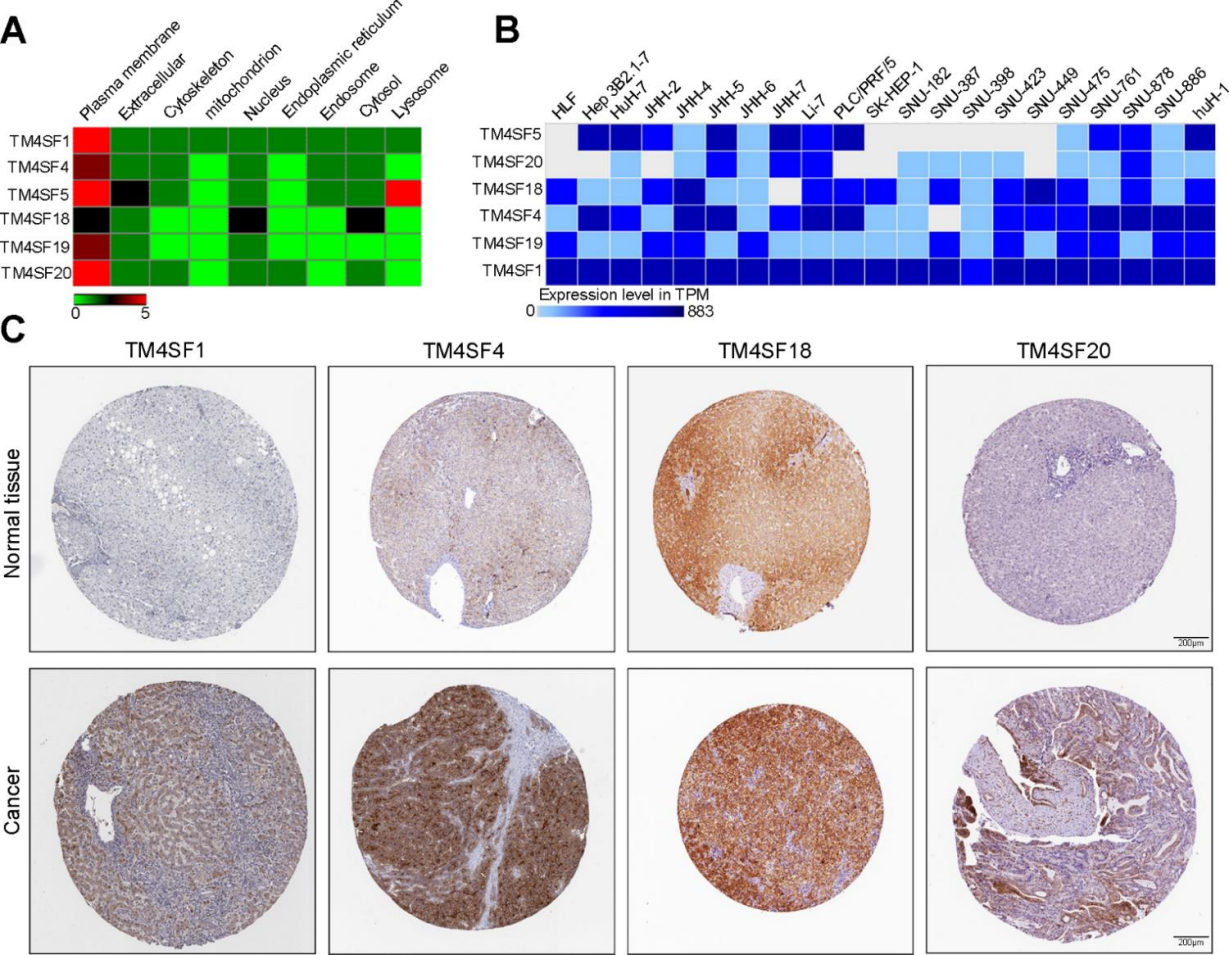
**The expression of TM4SF family members in LIHC cell lines and tissues.** (**A**) Localization of TM4SFs genes. (**B**) TM4SFs family members were distinctively expressed in LIHC cell lines. (**C**) Representative immunohistochemistry images of TM4SFs in HCC and normal liver tissues in the HPA database.

We then determined the expression of TM4SF family members in LIHC cells and tissues. According to the GeneCard, TM4SFs are abundant in the hepatic pancreas and predominantly localize to the cell plasma membrane. Additionally, TM4SF5 was expressed well in the lysosome, and TM4SF18 was located in the nucleus and lysosome (Figure 10A). Expression Atlas revealed that TM4SF1, TM4SF4, TM4SF18, and TM4SF19 were expressed in various cancer cell lines (Figure 10B). Furthermore, we investigated the protein levels of TM4SFs in LIHC using the HPA database and found that TM4SF1, TM4SF4, TM4SF18, and TM4SF20 were highly expressed in LIHC tissues. As shown in Figure 10C, the protein levels of TM4SF1 and TM4SF20 were overexpressed in LIHC patients with medium staining. TM4SF18 and TM4SF20 were remarkably up-regulated with solid staining. Overall, these results suggested that the TM4SF family members were all highly expressed in LIHC patients.

### TM4SF4 regulates tumor growth and stemness maintenance of CSCs in LIHC

**Figure 11:**
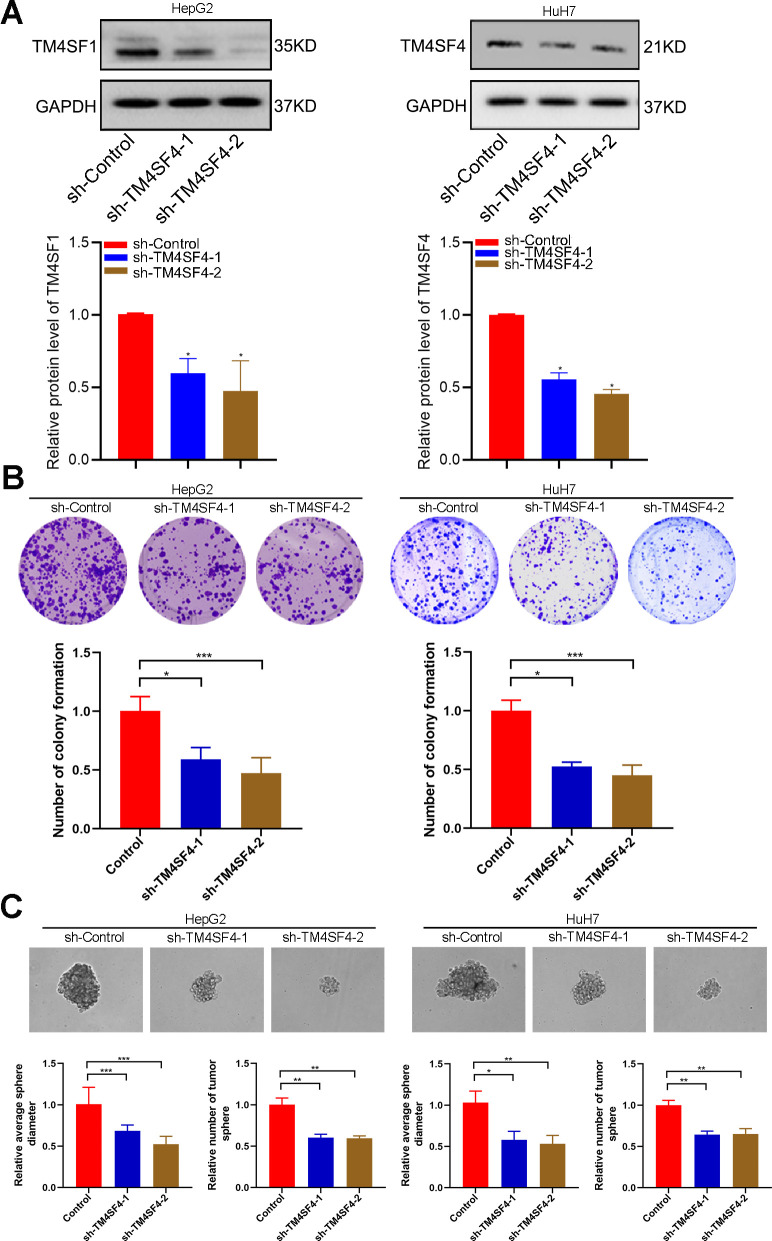
**TM4SFs regulate tumor growth and stemness maintenance of CSCs in LIHC.** (**A**) The levels of TM4SF4 transfection with shRNA were analyzed by WB. (**B**) Colony formation assays showed that knockdown of TM4SF4 inhibited CRC cell growth. (**C**) Sphere formation analysis suggested that the downregulation of TM4SF4 could significantly inhibit the sphere formation capacity. *p < 0.05, **p < 0.01, ***p < 0.001.

To probe the function of TM4SFs, we silenced the expression of these genes in HepG2 and HuH7 cells using targeted siRNA. Two shRNAs targeting the coding regions of TM4SF4 were tested for their knockdown efficiency (Figure 11A). Then, colony and sphere formation were applied to determine the effect of TM4SF4 on LIHC cells. The colony analysis suggested that the viability of clone formation was remarkably suppressed after TM4SF4 silencing (Figure 11B). Sphere formation analysis suggested that downregulation of TM4SF4 considerably inhibited the sphere formation capacity in LIHC cells (Figure 11C). These results suggested that TM4SF4 may play a significant role in the growth and stemness maintenance of LIHC cells.

### TM4SFs promote the migration and invasion of LIHC cells

**Figure 12:**
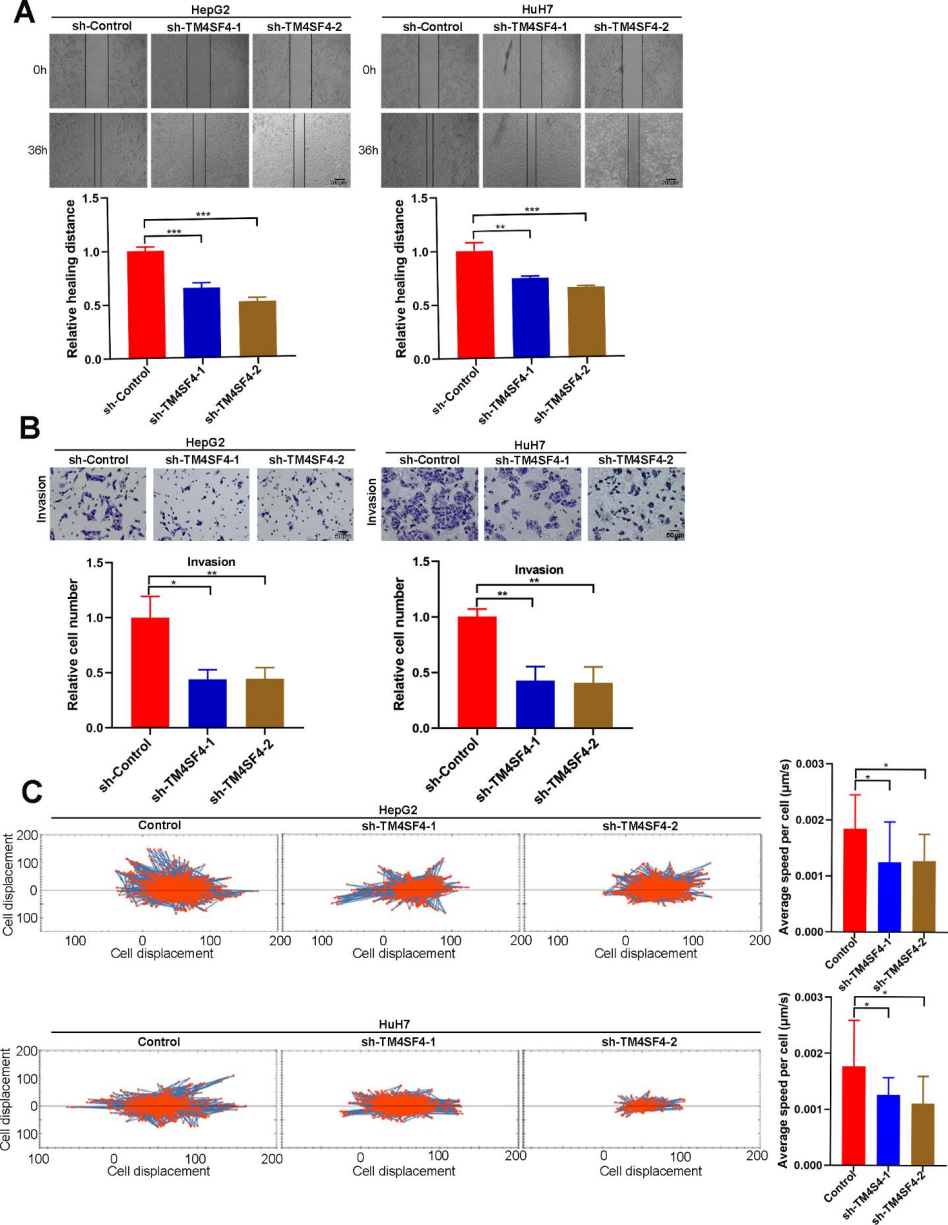
**TM4SF4 silencing suppressed the migration and invasion of LIHC cells.** (**A**) Scratch healing experiments were used to detect the migration ability of TM4SF4 silencing cells. (**B**) Transwell assays of cell invasion were performed to test the effects of TM4SF4 knockdown invasion of HepG2 and HuH7 cells. (**C**) Cell migration was assayed using a high-content imaging system and analyzed by the mean square displacement and speed. *p < 0.05, **p < 0.01, ***p < 0.001.

**Figure 13:**
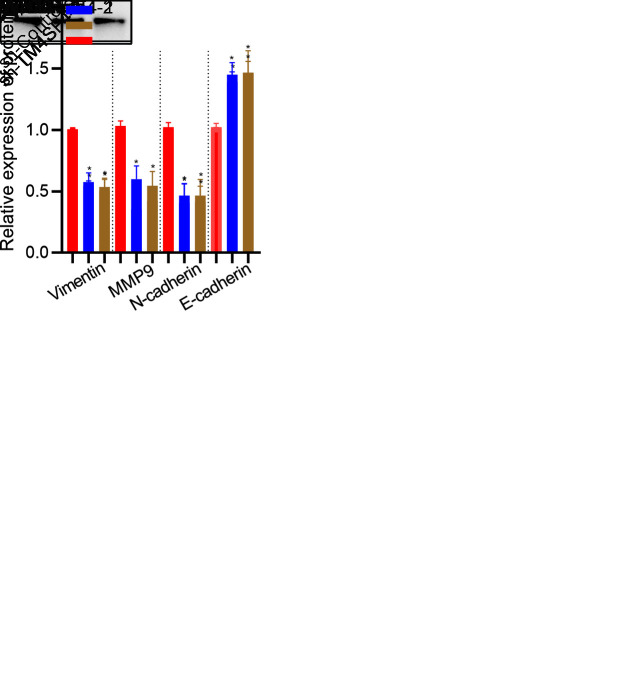
**TM4SF4 modulated EMT-associated gene expression.** (**A**) WB analyzed the expression of E-cadherin, Vimentin, N-cadherin, and MMP9.

Then, wound healing assay suggested that silencing TM4SF4 suppressed the cell healing ability (Figure 12A). Transwell assay revealed that TM4SF4 knockdown suppressed the invasion ability of HepG2 and HuH7 cells (Figure 12B). Subsequently, we investigated cell migration by High-Throughput Connotation System. We found that TM4SF4 knockdown cells exhibited a lower cumulative displacement than that of the control cells in 24h (Figure 12C). These cells moved considerably 2-fold slower on average (Figure 12D). WB analysis further confirmed that TM4SF4 knockdown remarkably down-regulated the protein level of MMP9, N-cadherin, and Vimentin. Conversely, E-cadherin was significantly up-regulated in the TM4SFs knockdown cells (Figure 13A).

## DISCUSSION

With the gradual increase in hepatocellular carcinoma incidence rates, LIHC ranks as the second leading cause of cancer-related mortality worldwide [18, 19]. It is essential to discover novel biomarkers and target treatments for LIHC. Bioinformatics analysis is a rapidly developing discipline with the characteristics of high efficiency that can be used to explore the predictive value of the TM4SFs in a short time [20, 21].

Numerous studies have reported that TM4SF family members play a crucial role in many kinds of cancer [22–24]. However, their biological roles and prognostic value in LIHC have rarely been characterized. We first demonstrated that TM4SFs were overexpressed in multiple cancer types, especially in LIHC tissues, compared to the corresponding normal tissues. High expression of TM4SF1 and TM4SF19 was positively correlated with tumor grade and TP53 mutation rate. It is generally thought that high-grade tumors are correlated with poor tumor differentiation, a high degree of tumor cell invasion, and more widespread lymphatic metastasis. TP53 is a tumor suppressor gene with a high frequency of mutation in tumors [25]. TP53 mutation may lead to the downregulation of the immune response and serve as a biological marker for prognostic evaluation in LIHC [26, 27]. Previous studies have shown that high expression of TM4SF1 was an independent risk factor for poor outcomes in lung cancer [28, 29]. Therefore, we investigated the associations between the expression of TM4SFs and prognosis in LIHC and found that TM4SF1, TM4SF5, TM4SF19, and TM4SF20 overexpression were considerably associated with poor prognosis. Furthermore, higher levels of TM4SF1 and TM4SF19 expression were remarkably correlated with poor RFS and PPS in LIHC. Therefore, TM4SF overexpression is involved in the tumor progression of LIHC.

Recently, numerous studies have confirmed that gene mutations and copy number variations are believed to be important drivers of tumorigenesis and the development of LIHC [30]. According to the cBioPortal database, we found that the copy number amplification of TM4SF1, TM4SF4, TM4SF18, TM4SF19, and TM4SF20 and the copy number deep deletion of TM4SF5 may be possible reasons for the abnormally high expression of the TM4SF gene family. Although the frequency of mutations was not high, patients with TM4SF4 and TM4SF18 mutations exhibited a markedly shorter OS than patients without the mutation, which suggested that these gene mutations were associated with a negative prognostic impact. Epigenetic alterations have been established as hallmarks of tumorigenesis and metastasis [31]. Aberrant DNA methylation is a potential reversible therapeutic target due to the relative stability of these alterations [32, 33]. It has been reported that the promoter methylation levels of AKT3, CD147, LINE1, MAGEA1, RASSF1A, and SFRP1 were considerably correlated with OS, tumor volume, and cancer properties [34, 35]. Our findings showed that TM4SF methylation levels in cancer were considerably lower than those in adjacent normal tissue. The methylation levels of these genes’ promoters showed an apparent negative correlation with gene expression levels, which suggested that promoter hypomethylation of TM4SFs remained the primary driver of high expression of these genes.

Previous studies have revealed the underlying mechanism by which TM4SFs are involved in the malignant progression of cancers [5, 36]. To further investigate the underlying mechanism responsible for TM4SF family member regulation, we performed gene enrichment analysis of the core genes (protein interactions, co-expressed genes) potentially correlated with TM4SF function. The results revealed that these core genes were mainly involved in the positive regulation of cell migration, acute inflammatory response, mesenchymal cell differentiation, cell-substrate adhesion, regulation of MAP kinase activity, and the PI3K-Akt signaling pathway, which is in line with previous studies. Lekishvili reported that TM4SFs could interact with integrins, immunoproteins, and PDZ-domain-containing proteins and then form tetraspanin-enriched microdomains (TERMs), which could modulate cell migration, invasion, adhesion, and metastasis [5]. Huang et al. suggested that TM4SF1 could enhance cell migration, growth, and metastasis in liver cancer [6]. Xiaoqin Du reported that microRNA-520f suppressed cell migration and metastasis by targeting TM4SF1, and restoration of TM4SF1 could markedly abolish miR-520f-mediated cell migration and invasion via the PI3K/AKT and p38 MAPK signaling pathways [37]. Jung Weon Lee reported that TM4SF5 could drive EMT, which in turn contributes to drug resistance, enhanced invasion, and metastasis. They also suggested that strategies for anti-TM4SF5-related protein networks potentially reverse the fibrotic, tumorigenic, and tumor-maintaining functions in TM4SF5-overexpressing hepatic cells [10]. To further determine the role of TM4SFs in LIHC, we performed loss-of-function studies and found that TM4SF4 could modulate the migration and invasion ability via activating EMT in LIHC. Therefore, TM4SFs can be invoked as a reliable prognostic marker for LIHC.

## CONCLUSIONS

In summary, this study clarified the expression profile and predictive value of TM4SFs in LIHC patients. High expression and hypomethylation of TM4SF1, TM4SF19, and TM4SF20 were related to poor survival in LIHC patients. The model constructed based on the TM4SF-based signature could predict the long-term survival and response to chemotherapy and immunotherapy for LIHC patients. Finally, we performed colony formation, sphere formation, and Transwell assays and found that TM4SF4 knockdown could significantly suppress the growth, migratory, and invasive abilities of LIHC cells. Therefore, targeting TM4SFs will contribute to effective immunotherapy strategies and improve the prognosis of LIHC patients.

## MATERIALS AND METHODS

### Exploring differential TM4SFs expression, genomic alterations, and promoter methylation in LIHC

To identify the expression of TM4SFs in 33 cancer types (TCGA datasets), we analyzed the expression profiles with the Oncomine database. The transcriptome data and promoter methylation levels of the TM4SFs in LIHC were downloaded from HCCDB. Kaplan-Meier plots were used to determine the prognostic value of TM4SFs. The genomic variation, mutation, and prognostic data were downloaded from the cBioPortal database.

### Construction of a risk signature and evaluation of the predictive ability

To quantify gene expression profiles of LIHC patients, the random forest method was adopted to build a classification model named the TM4SFs-based score. The prediction model was assessed using the formula TM4SFs risk score = S (Coef i × Exp i). Based on the results of the gene signature, we performed a K-M survival analysis and ROC curve to illustrate the model’s performance. A nomogram was used to visualize the predictive model using RStudio, and the nomogram discrimination and accuracy were illustrated by ROC and calibration curves.

### Characterization of immune infiltrates between the risk groups

Then, we analyzed the association of risk score and the extent of immune cell infiltration in LIHC patients. The CIBERSORT algorithm was applied to analyze the cell infiltrations. To compare the differences between the groups, the Wilcoxon rank-sum test was applied.

### Prediction of the sensitivity of patients to immunotherapy/chemotherapy

We investigated the patient response to immunotherapy using the IMvigor-210 cohort. The response was divided into four response categories: partial and complete response (PR, CR), disease progressive or stable (PD, SD). Differences between responders and non-responders were analyzed using the Mann-Whitney U test. The “pRRophetic” was adopted to calculate the IC50 value of widely used chemotherapy drugs for each patient.

### Western blotting

Cells were lysed by the SDS buffer with phosphatase inhibitor on ice. Protein samples were loaded and separated on 4-12% SDS-PAGE gels and then transferred onto PVDF membranes. The transferred membranes were blocked with skimmed milk and incubated in primary antibodies. Finally, the membranes were incubated in secondary antibodies. Immunoreactive proteins were detected using a chemiluminescence solution.

### Cell culture and transfection

For cell-based experiments, liver cancer cell lines, including HepG2 and HuH7, were purchased from the Cell Bank of the Chinese Academy of Sciences (China, Shanghai). All cell lines were authenticated prior to use and routinely tested by DNA analysis. Cells were cultured in DMEM with 10% FBS. ShRNA targeting TM4SF4 was purchased from HedgehogBio, Inc. Transfection was performed using the Lipofectamine® 3000 kit according to the manufacturer’s protocol.

### Colony formation

500-800 liver cancer cells were inoculated into a six-well plate. Every three days, the culture medium was replaced with a fresh medium. After 7-10 days, the six-well plate was removed and washed 1-2 times with sterile PBS. Excess PBS was aspirated using a pipette, and 500 μl of 4% paraformaldehyde was added for fixation for 20 minutes. The fixation solution was then discarded, and 1 ml of 1% crystal violet solution was added for staining for 20 minutes. The crystal violet staining solution was collected, and each well was gently rinsed with running water to remove excess dye, allowing it to air dry naturally. The plates were photographed, and the colonies were quantified to determine the clonogenic ability.

### Automated cell tracking

Live cell imaging was conducted with an Operetta high-content imaging system with temperature and CO_2_ control settings at 37° C and 5% CO_2_. LIHC cells were seeded in 24-well plates containing 100 μl cell culture medium. Then, the 24-well plates were transferred to the preheated Operetta system for an additional 30-minute incubation. Subsequently, digital phase contrast images were captured at 10X magnification. Image acquisition continued for up to 16 hours at 15-minute intervals. Image segmentation was performed using the Find Cells function within the Harmony software, which employs a dedicated algorithm for segmenting digital phase contrast images.

### Transwell assays

Transwell assays were performed to determine the invasion and migration ability. LIHC cells (3 × 10^5^) were seeded into the top chamber with FBS-free medium. Medium with 10% FBS was added to the lower chamber. Cells on the lower membrane were fixed with formaldehyde and stained with crystal violet; images of different areas (six random fields) were counted after 24h. The upper chamber was pre-coated with Matrigel for the invasion assay.

### Wound healing

LIHC cells (3 × 10) were cultured in 6-well plates. The scratch wound was built with a 10-μl pipette tip after the fusion degree of cells reached 90% confluence. The complete medium was replaced with FBS-free medium. After 24-36 h, the scratch zones were photographed by inverted microscopy. Each experiment was repeated three times or more.

### Availability of data and materials

Authors can provide all data sets analyzed during the study on reasonable requirements.

### Consent for publication

All authors have agreed to publish the article.

## Supplementary Material

Supplementary Figures
