# Supplementary material for: Integrated multi-omics analyses reveal the TM4SF family genes with prognostic and therapeutic relevance in hepatocellular carcinoma
Source: Aging (Albany NY). 2024 Jan 10;16(1):593–616. doi: 10.18632/aging.205398 (PMC10817404; doi:10.18632/aging.205398)
Supplement: Supplementary Figures [file aging-16-205398-s001.pdf]

SUPPLEMENTARY FIGURES

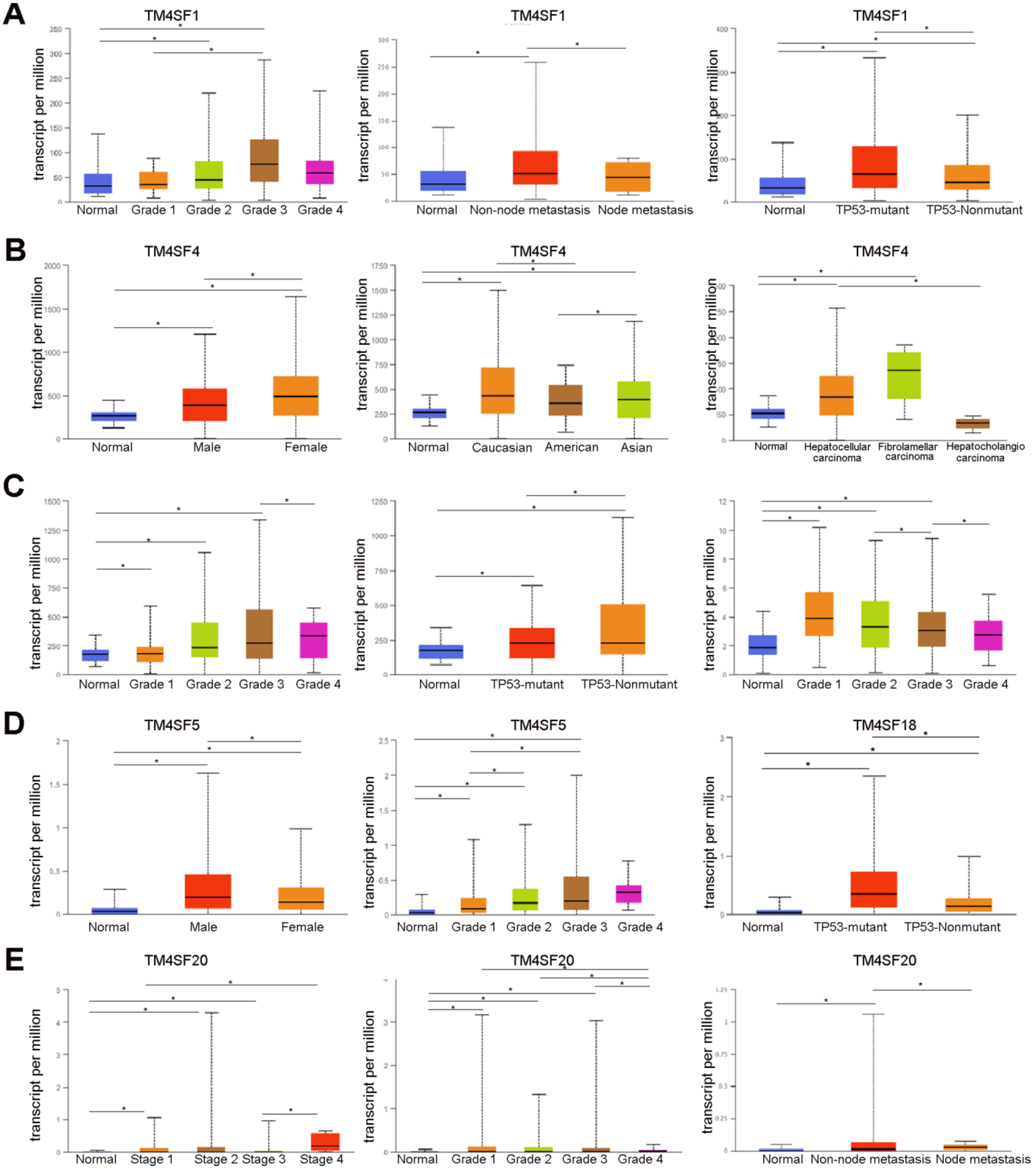

Supplementary Figure 1. Analysis of the relationship between TM4SFs expression and clinicopathologic parameters in LIHC (**A**–**E**).

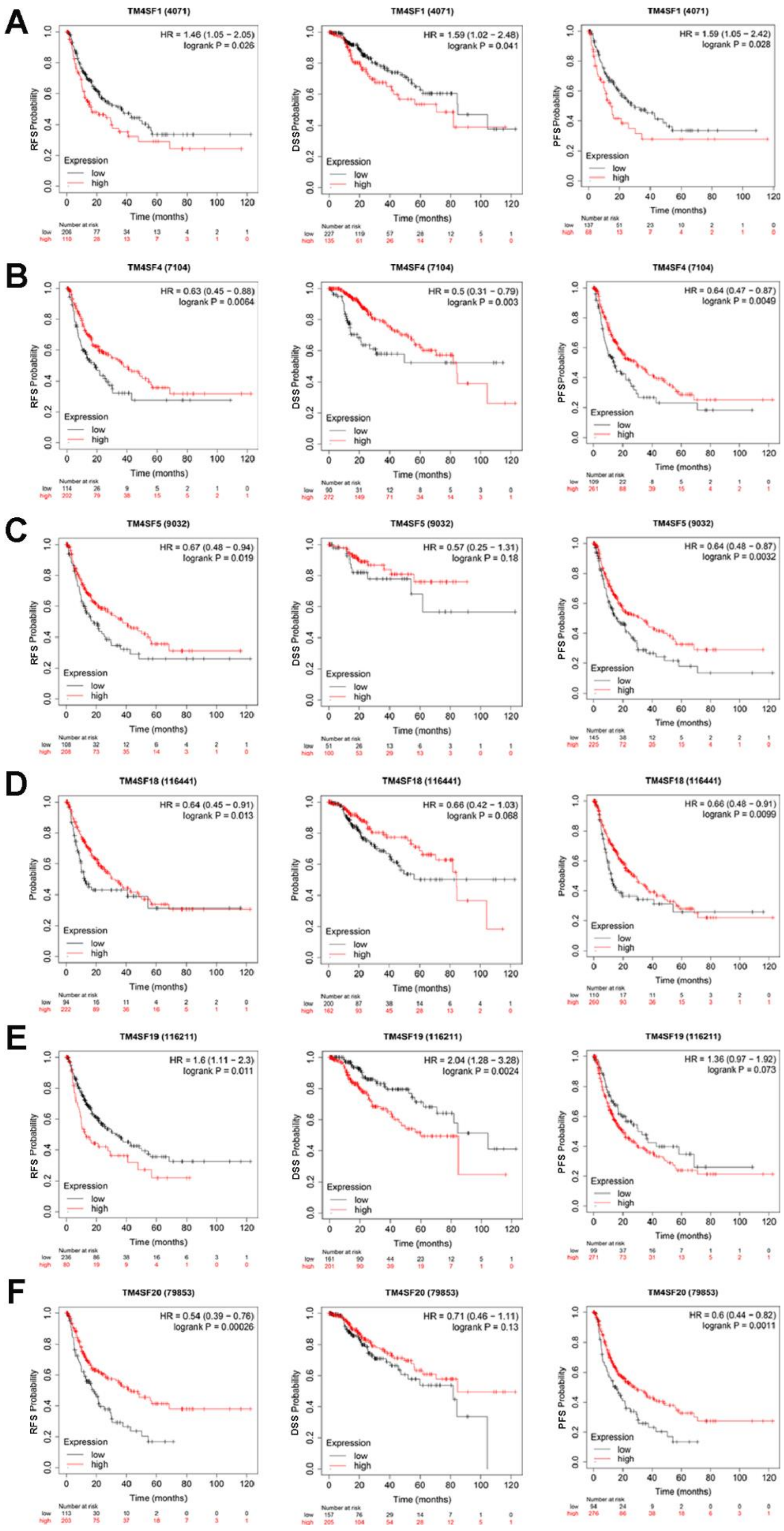

Supplementary Figure 2. Kaplan–Meier survival curve analysis for TM4SF family members in LIHC. (**A**) TM4SF1, (**B**) TM4SF4, (**C**) TM4SF5, (**D**) TM4SF18, (**E**) TM4SF19, (**F**) TM4SF20.

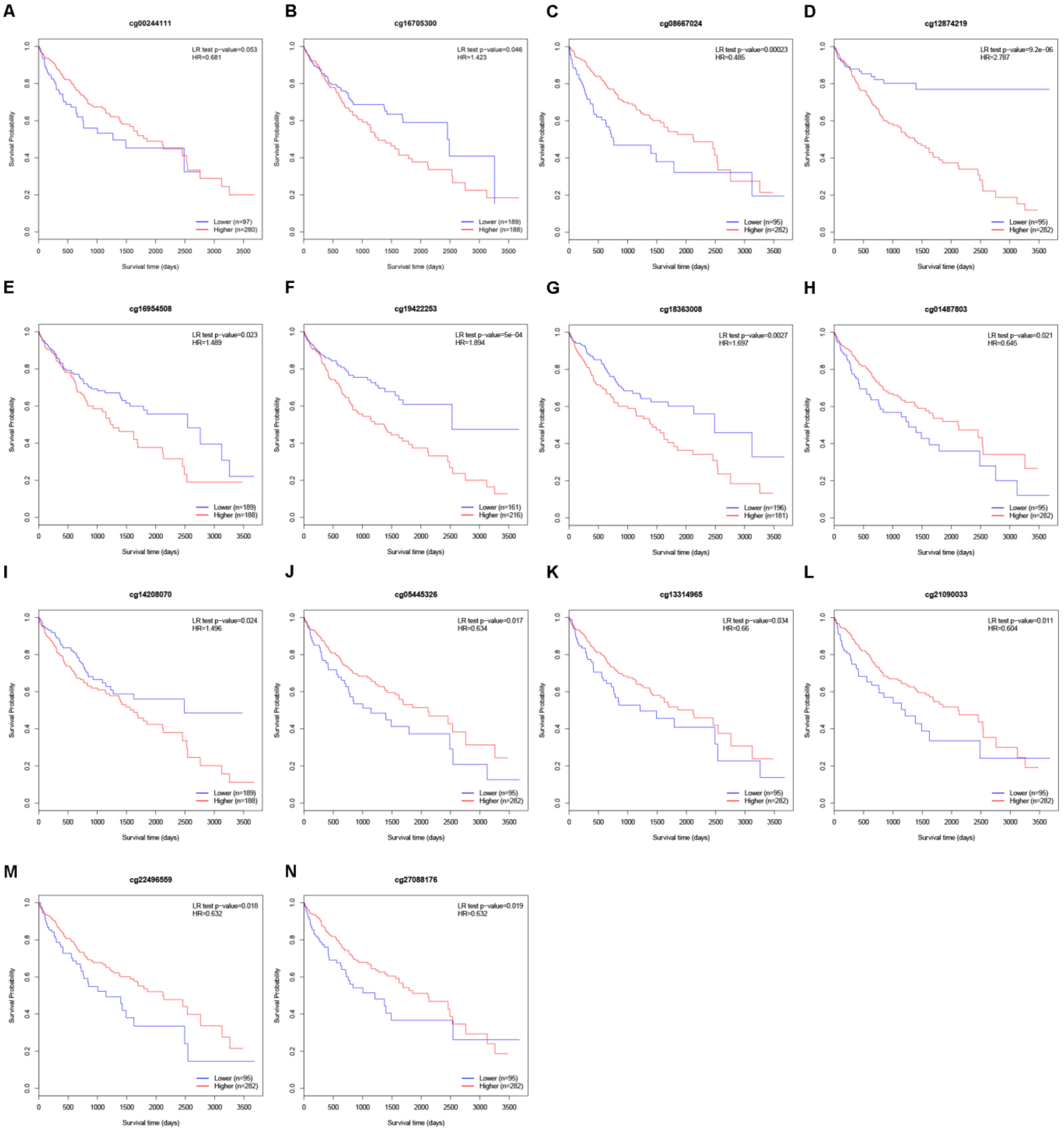

Supplementary Figure 3. Prognostic value of TM4SF-specific CpG site methylation in LIHC (**A**–**N**).
